# Characterization of the complete mitochondrial genome of Dongyangjiang White-toothed Shrew, *Crocidura dongyangjiangensis* (Eulipotyphla: Soricidae) and its phylogenetic analysis

**DOI:** 10.1080/23802359.2021.1893617

**Published:** 2021-03-18

**Authors:** Haotian Li, Guochen Zhang, Guanze Liu, Xiaoqing Sun, Xinghan Lin, Yaoyao Li, Yuchun Li

**Affiliations:** Marine College, Shandong University, Weihai, China

**Keywords:** Complete mitochondrial genome, *Crocidura dongyangjiangensis*, phylogenetic analysis

## Abstract

The complete mitochondrial genome of the Dongyangjiang White-toothed Shrew (*Crocidura dongyangjiangensis*), a newly discovered *Crocidura* species, is sequenced and characterized. The total length of the genome is 16,883 bp, and has similar base composition and gene arrangement to other vertebrates. It contains 13 protein-coding genes (PCGs), two ribosomal RNA (rRNA), 22 transfer RNA (tRNA), a replication origin (OL) and a control region. The phylogenetic tree shows that *C. dongyangjiangensis* was fully resolved in a clade with three other species of *Crocidura* and it has a sibling relationship with *Crocidura tanakae*. This analysis reveals the evolutionary relationship of 16 *Crocidura* species.

The Dongyangjiang White-toothed Shrew *Crocidura dongyangjiangensis* Liu Y, 2019 belongs to the family Soricidae. This species is a relatively small *Crocidura* species recently discovered in southeast China (Liu et al. 2019). Due to their small size and weak migratory ability, *Crocidura* species are easily affected by topographic complexity and climatic oscillations, and may have undergone a rapid and recent radiation (Giarla and Esselstyn 2015). It is difficult to reveal the correct phylogenetic relationship of this genus based on a few gene fragments (Chen et al. 2020). Therefore, in this study, we sequenced and described the mitochondrial genome of *C. dongyangjiangensis*, and provide information for further molecular studies of this species.

The voucher specimen captured from Jinhua Mountains in Zhejiang Province (120°31′E, 29°12′N), which is adjacent to its type locality (119°38′E, 29°12′N). The voucher number of this specimen is S3990 (URL, Li Yuchun and li_yuchun@sdu.edu.cn), and stored in Shandong University (Weihai). Total genomic DNA was extracted from muscle tissue using the Ezup Column Animal Genomic DNA Purification Kit (Sangon Biotech Co., Ltd., Shanghai, China). Whole mitochondrial genome was amplified with 17 pairs of primers (T1–T17) designed by our laboratory based on the mitochondrial genomes of *Crocidura fuliginosa* (Li et al. 2019). The 17 pairs of primers are T1F 5′-TCACTCTTCTCGCTCCG-3′ and T1R 5′-GTGCTTATTAACTTGGGTT-3′; T2F 5′-CACCCCCACGGGAAACAGC-3′ and T2R 5′-ACGCTTTCTTTATTGATGGCTGC-3′; T3F 5′-CCTGGTGATAGCTGGTTA-3′ and T3R 5′-CAATAGGTTGAAGGAGGC-3′; T4F 5′-CAGGATTCGTTAGAGTGG-3′ and T4R 5′-TGATTTCTCAAGGGTAGG-3′; T5F 5′-CCCACTGACCCGAACTAT-3′ and T5R 5′-GGGGAAGGTATTAACCGA-3′; T6F 5′-CTAGAATTATAGGAATTGAACCTAC-3′ and T6R 5′-TGAAGCCAGTTGGTTAGG-3′; T7F 5′-ACAGACCGAGAGCCTTCA-3′ and T7R 5′-GGGAATAGCCTGTAAATAAG-3′; T8F 5′-ATTCCTACGGGAGTAAAAGC-3′ and T8R 5′-TCTTTAACTTAAAAGGTAAACGCT-3′; T9F 5′-GAAGTAGATAATCGCCCAGT-3′ and T9R 5′-TAAAATCAAATCATATTACTAAGCC-3′; T10F 5′-TAATCACCCACCAAACTCAAGC-3′ and T10R 5′-TAATGAGTCGCAATCAATGGTTTT-3′; T11F 5′-TTATTCAAACACCCTGATGCT-3′ and T11R 5′-TCTGTAGCGGAGAAAGTTATGAT-3′; T12F 5′-CAATGCTAAAACTTATTATTCCTTC-3′ and T12R 5′-ATAATAGTTGGACTGTGAATGCGTT-3′; T13F 5′-ATAGGAGCCACAGCACTAATAAT-3′ and T13R 5′-TGATTCCTAATACCAACGGATAACT-3′; T14F 5′-CGAGAAAGACTGCAAGAA-3′ and T14R 5′-GTAAACGGCTGTTAGGGT-3′; T15F 5′-CCCAAAATGATAGCAAAAAAAT-3′ and T15R 5′-ATCAACGAGGATTTTTCTTGTCAT-3′; T16F 5′-CCATAAATAGGTGAGGGTT-3′ and T16R 5′-AATATCAGCTTTGGGTGC-3′; T17F 5′-CTCGTCTTGTAAAGCAGAAATGGA-3′ and T17R 5′-CCATAGAGATGTCGTATTTCAGAGG-3′. The resulting sequences were assembled, annotated and analyzed using the BioEdit 7.2.5 (Hall 1999), MITOS 2 (Bernt et al. 2013) and MEGA X (Kumar et al. 2018) software, respectively. We performed the maximum likelihood (ML) phylogenetic tree with the GTR + I + G model using MEGA X software (Kumar et al. 2018) based on the 13 protein-coding genes (PCGs) of 16 *Crocidura* species, with *Suncus murinus* designated as the outgroup. The bootstraps were obtained using a rapid bootstrapping algorithm with 1000 replicates.

The complete mitogenome of *C. dongyangjiangensis* is deposited in GenBank under the accession number MW376861, and has a total length of 16,883 bp, and is similar in base composition and gene arrangement to other vertebrates. The overall base composition is A (32.88%), T (31.76%), C (22.25%), and G (13.10%). It contains 37 genes, including 13 PCGs, two ribosomal RNA (rRNA) genes, 22 transfer RNA (tRNA) genes. In addition, there is a replication origin (OL) with a length of only 37 bp and a control region with a length of 1420 bp, located between the tRNA-Asn and tRNA-Cys genes and between the tRNA-Pro and tRNA-Phe genes, respectively. Most genes are encoded on the heavy strand, except for the nicotinamide adenine dinucleotide dehydrogenase subunit 6 (ND6) and eight tRNA genes (tRNA-Gln, Ala, Asn, Cys, Tyr, Ser, Glu, and Pro) which are encoded on the light strand. All PCGs initiate with ATG except for ND2, ND3, and ND5, which began with ATC, ATT and ATA. Twelve PCGs terminated with TAA, TA–, and T––, whereas the *Cyt b* gene terminated with AGA. The lengths of the 22 tRNA genes vary from 59 to 75 bp. In addition, overlap and noncoding bases in the mitogenome are also observed.

Phylogenetic analysis of mitochondrial genomes of representative *Crocidura* species fully resolved *C. dongyangjiangensis* in a clade with *Crocidura tanakae*, *Crocidura attenuate* and *Crocidura lasiura* (Figure 1). The data show that *C. dongyangjiangensis* has a sibling relationship to *C. tanakae*, although this relationship is not supported. The *Crocidura* species included in this phylogenetic tree are divided into four clades, from the root to the terminal clade of the tree, they are *Crocidura russula* mainly distributed in western and southern Europe; *Crocidura sibirica* and *Crocidura shantungensis* mainly distributed in central and eastern Asia; five *Crocidura* species in northeast and southern China; and the remaining eight *Crocidura* species distributed in Southeast Asia and South Asia.

**Figure 1.**
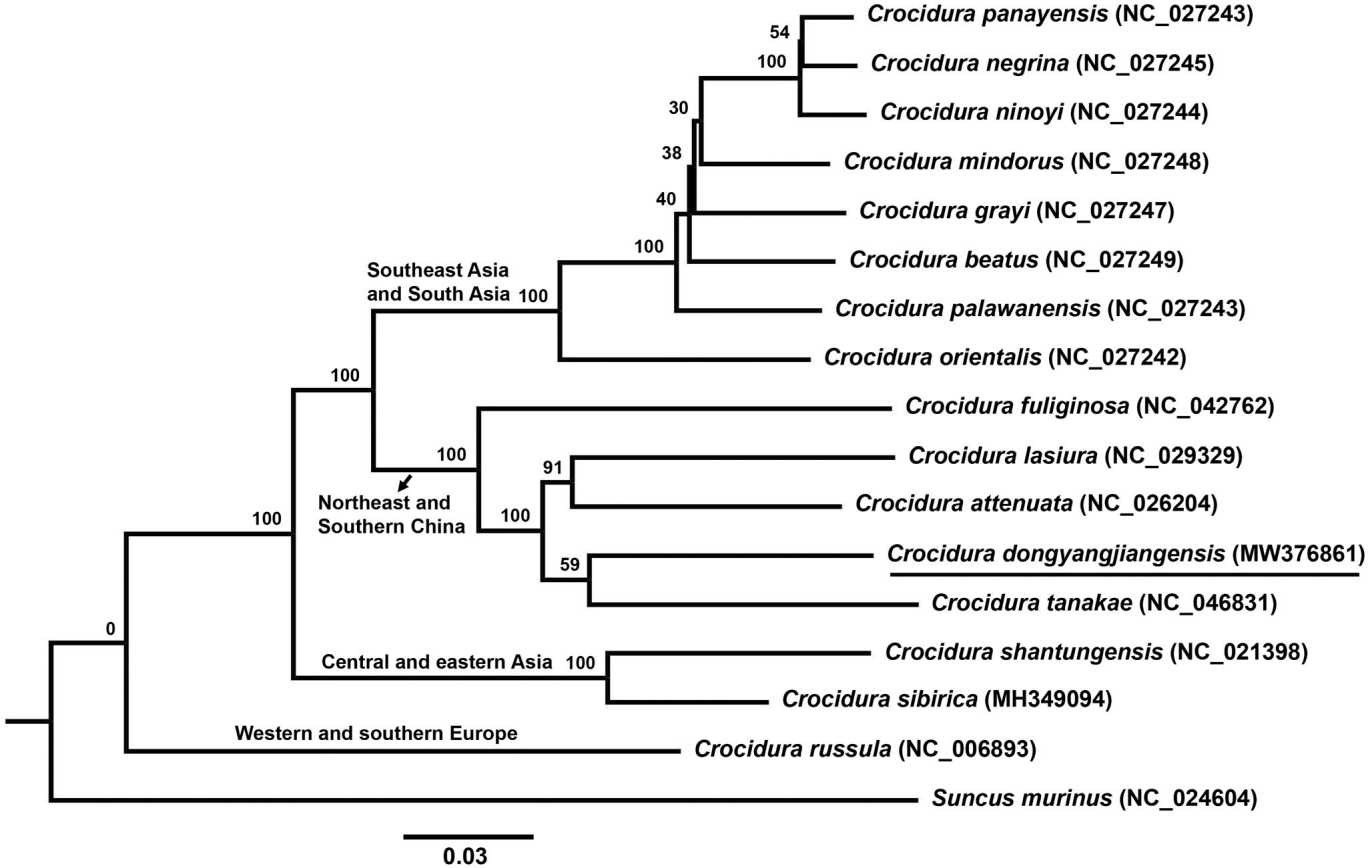
ML phylogenetic tree of *Crocidura* species based on 13 PCGs under GTR + G + I model. Nodal support was estimated by 1000 bootstrap replicates. ML bootstrap values are shown above the nodes.

The complete mitochondrial genome of *C. dongyangjiangensis* and the phylogenetic result provide the molecular data and genetic information for species delimitation, phylogenetic relationship and evolutionary history of *Crocidura* species.

## Data Availability

The data that support the findings of this study are openly available in GenBank at https://www.ncbi.nlm.nih.gov/, reference number MW376861.
